# Publishing on Policy: Trends in Public Health

**Published:** 2010-12-15

**Authors:** Amy A. Eyler, Mariah Dreisinger

**Affiliations:** Prevention Research Center in St. Louis, George Warren Brown School of Social Work, Washington University in St. Louis; Washington University in St. Louis, St. Louis, Missouri

## Abstract

Our goal was to explore the number and topics of policy articles published in general public health journals. We conducted an audit of articles in 16 public health journals from 1998 through 2008. Results showed no trends for the decade studied; only 3.7% of all articles published in these journals were policy-related, and the topics most represented were smoking/tobacco, health care, and school policy. As policy research on public health issues continues to develop, researchers have an opportunity to increase dissemination through publication in general public health journals.

## Objective

Policy approaches to improving health are designed to provide opportunities, support, and cues to help people develop healthier behaviors (1) by initiating changes in physical, economic, or social environments (2). In both research and practice, focus has increased on policy approaches to health promotion (3). The purpose of our study was to explore policy research published in a sample of general public health journals during the last decade.

## Methods

To develop the list of journals for our review, the research team brainstormed a list of public health journals that cover a broad number of topics. We then supplemented this list by looking at impact factor lists and core public health journal lists on the Internet. Because the focus of this study was to identify policy articles in broad-topic public health journals, we did not include journals specific to a single topic such as those addressing health policy, medicine, nutrition, or disease. Journals selected also had to have been published throughout the time studied. We chose 16 general public health journals (Appendix) for the study.

We defined a policy article as one focusing on a policy, law, regulation, or rule. This included articles describing formal policies such as taxation and legislation as well as less formal policies such as workplace or school wellness. Articles about the built environment without mention of policy were excluded.

Our goals were to count the number of policy articles published by topic and to assess trends over time. Using online archives, we conducted a systematic audit of articles published from January 1, 1998, to December 31, 2008, by collecting tables of contents from each journal issue in the study time frame. We reviewed each table of contents and compiled a list of policy articles. Commentaries on policy topics that included references were included in our count. If the policy content was unclear from the title of the article, we searched the abstract or full text. We discussed any articles in question before including or excluding them. We also counted the total number of articles the journals published each year.

We sorted the policy articles by journal, year of publication, and topic. The categories for the policy topics were developed a priori and amended during the sorting process. We included all school policies regardless of topic (eg, nutrition, health) in an umbrella school policy category. We placed any policy taking place outside the United States in an international policy category, which we included in the overall sample and analyzed separately from the total sample.

## Results

**Figure 1:**
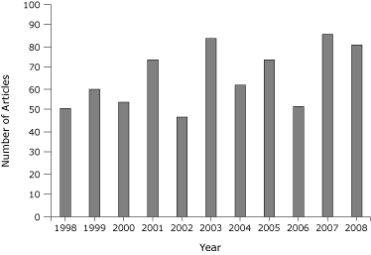
Total policy articles published in 16 general public health journals, by year, 1998-2008. The journals are listed in the Appendix.

**Figure 2:**
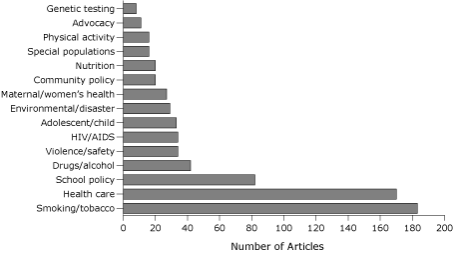
Number of articles published on policy topics in 16 general public health journals, 1998-2008. The journals are listed in the Appendix.

Of 19,438 articles published during 1998-2008, 725 (3.7%) were related to policy (Figure 1). The percentage of policy articles published per year in the 16 journals ranged from 4.7% in 2001 and 2003 to 2.7% in 2006. The *Journal of School Health*, *American Journal of Health Promotion*, and *American Journal of Public Health* had the highest percentage of policy articles during the study period. Of the 725 articles, 149 were international policy articles. The most common policy topic was smoking/tobacco (n = 183) (Figure 2), which showed an overall increasing trend during the 10-year period: 14 articles in 1998 increased to 24 articles in 2008. Health care was the second-most frequent policy topic (n = 170) followed by school policy (n = 82). School policy articles also showed an increasing trend over time.

**Table:** 

**Year**	**Number of Articles**
1998	51
1999	60
2000	54
2001	74
2002	47
2003	84
2004	62
2005	74
2006	52
2007	86
2008	81

**Table:** 

**Topic**	**Number of Articles**
Genetic testing	8
Advocacy	11
Physical activity	16
Special populations	16
Nutrition	20
Community policy	20
Maternal/women’s health	27
Environmental/disaster	29
Adolescent/child	33
HIV/AIDS	34
Violence/safety	34
Drugs/alcohol	42
School policy	82
Health care	170
Smoking/tobacco	183

The topics of maternal/women's health and drugs/alcohol both showed slight reverse time trends; more articles were published in the earlier years than in the later years studied. Physical activity and nutrition policy articles (not related to school policies) were 2 of the least prevalent topics (16 and 20 articles, respectively).

We analyzed the international policy articles separately; the most frequent topics were health care (n = 59) and smoking/tobacco (n = 37). Drugs/alcohol and adolescent/child policy articles were next (n = 9). There was no trend in number of international policy articles published by year; the highest number was in 2006 and the lowest, in 2000.

## Discussion

Changes in policy can help foster and maintain individual-level behavior change (4). Interventions focused solely on individual-level health changes are overall minimally effective and can be costly, and the changes in health may be less sustainable than policy and environmental approaches (1,3,4). In contrast to interventions focused on individuals, policies have the potential to affect health across populations (2).

Despite enhanced interest in policy interventions for health improvement (1,3,5), the number of policy articles published in general public health journals has remained steady during the past decade. Two topics that were most prevalent in the audit (smoking/tobacco and health care) have direct policy implications and a long history of policy development and implementation. School policy was also well represented in the study, probably because schools are a setting governed by federal, state, and local policies. In particular, the federal mandate for school wellness policies may have contributed to the number of school policy articles (6).

Limitations of this study should be noted. Even though publications may not have increased over time, other avenues of dissemination such as non–peer-reviewed briefs and reports were not counted in this audit. Additionally, researchers may be more inclined to submit policy-focused articles to health policy journals that were intentionally not included in our study.

Policy interventions have made vital contributions to health achievements. Examples include school vaccination laws that helped reduce the rates of infectious disease and tobacco control laws that helped reduce the rates of chronic disease (7,8). More recently, policies are seen as a way to influence population rates of other health problems such as physical inactivity and obesity. As policy research on these topics continues to develop, researchers have an opportunity to increase dissemination through publication in general public health journals.

## Appendix. List of Journals Reviewed for Policy Articles

- *American Journal of Behavioral Health*
- *American Journal of Health Promotion*
- *American Journal of Preventive Medicine*
- *American Journal of Public Health*
- *Australian and New Zealand Journal of Public Healt*h
- *Community Health*
- *Health Education and Behavior*
- *Health Education Research*
- *Health Promotion International*
- *Health Psychology*
- *Journal of Behavioral Medicine*
- *Journal of School Health*
- *Journal of Public Health Practice and Management*
- *Preventive Medicine*
- *Public Health Reports*
- *Social Science and Medicine*
